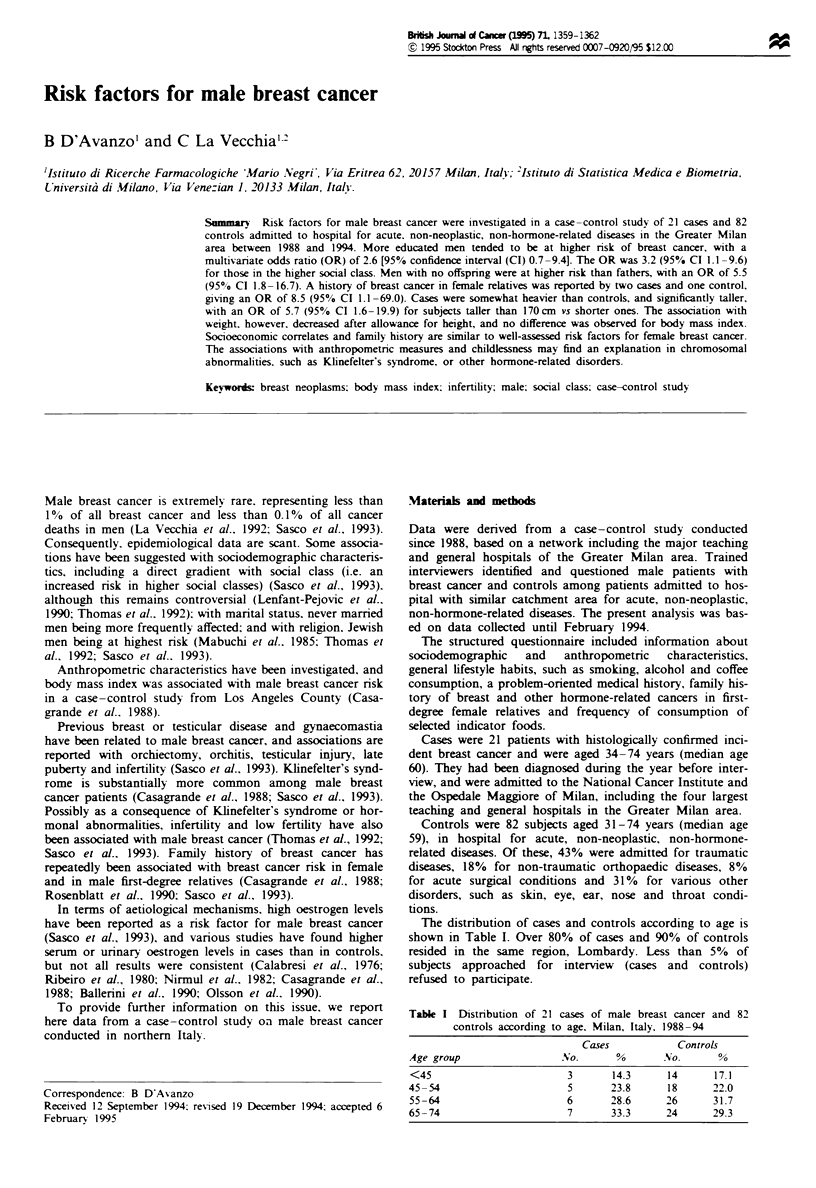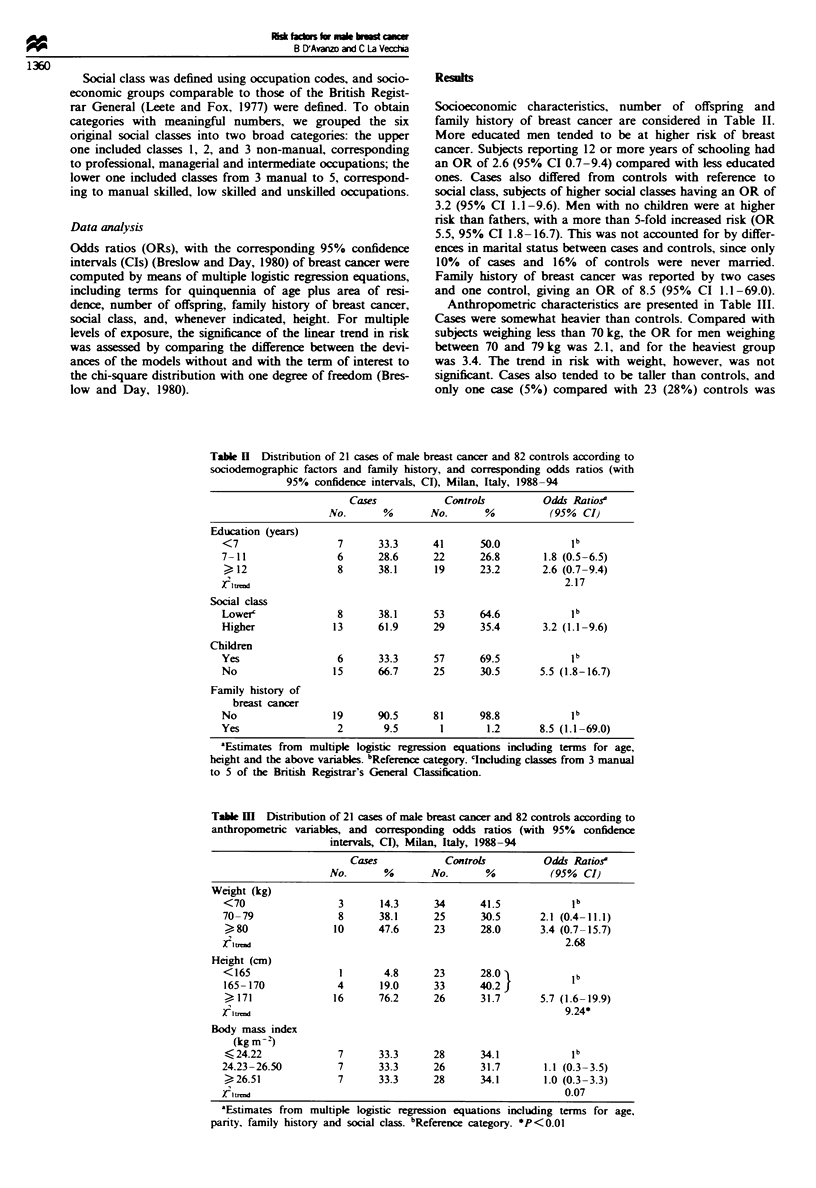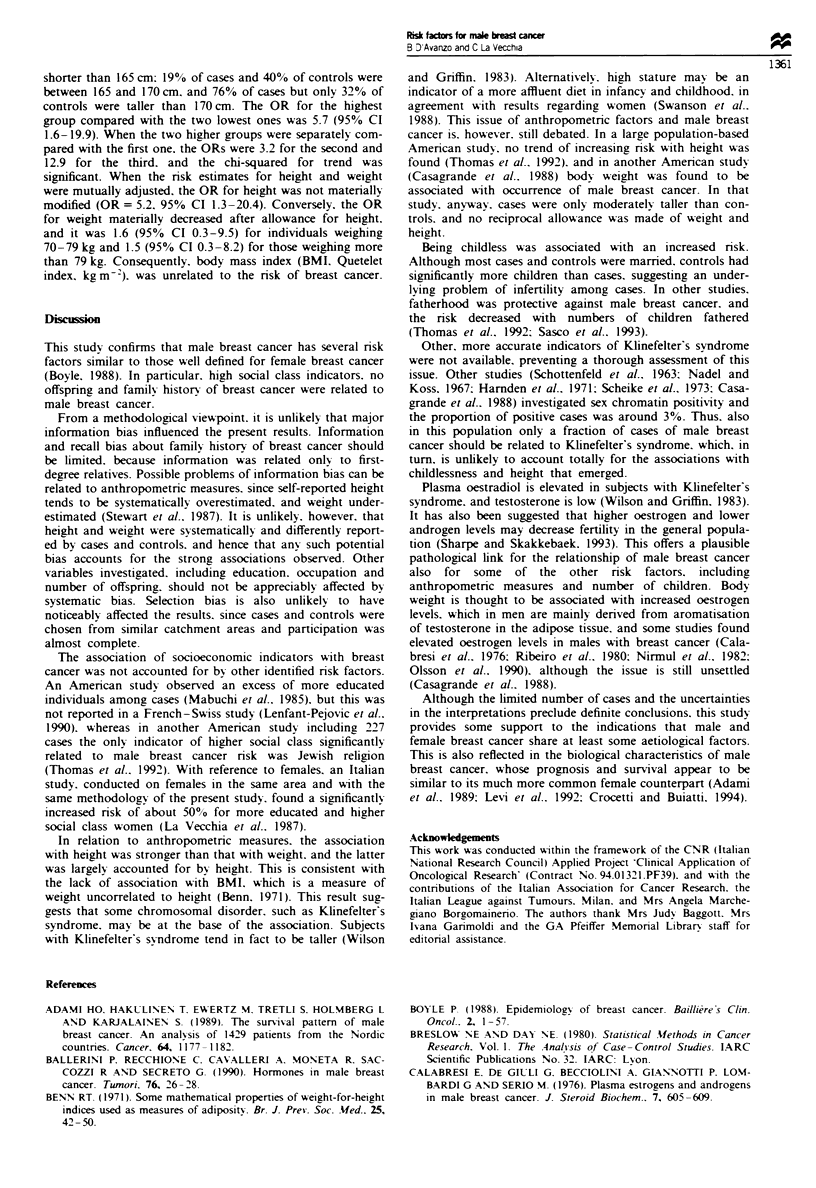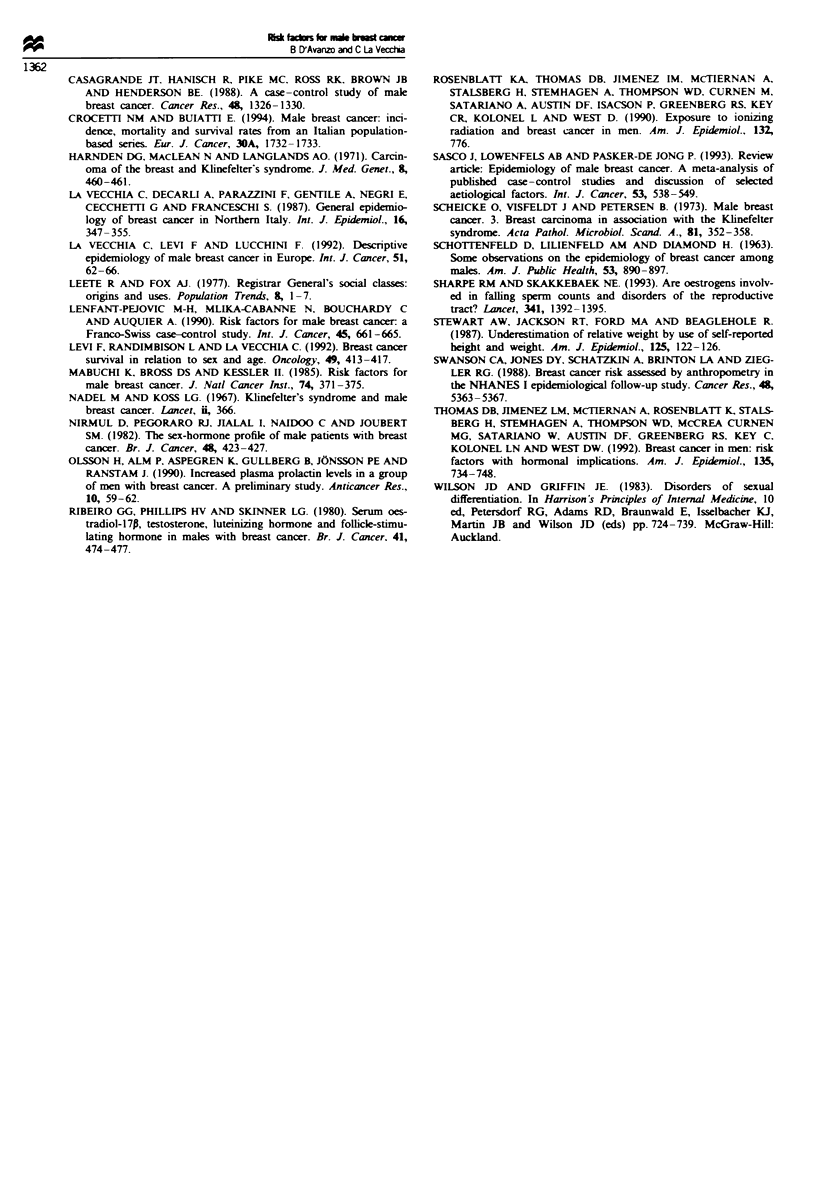# Risk factors for male breast cancer.

**DOI:** 10.1038/bjc.1995.264

**Published:** 1995-06

**Authors:** B. D'Avanzo, C. La Vecchia

**Affiliations:** Istituto di Ricerche Farmacologiche Mario Negri, Milan, Italy.

## Abstract

Risk factors for male breast cancer were investigated in a case-control study of 21 cases and 82 controls admitted to hospital for acute, non-neoplastic, non-hormone-related diseases in the Greater Milan area between 1988 and 1994. More educated men tended to be at higher risk of breast cancer, with a multivariate odds ratio (OR) of 2.6 [95% confidence interval (CI) 0.7-9.4]. The OR was 3.2 (95% CI 1.1-9.6) for those in the higher social class. Men with no offspring were at higher risk than fathers, with an OR of 5.5 (95% CI 1.8-16.7). A history of breast cancer in female relatives was reported by two cases and one control, giving an OR of 8.5 (95% CI 1.1-69.0). Cases were somewhat heavier than controls, and significantly taller, with an OR of 5.7 (95% CI 1.6-19.9) for subjects taller than 170 cm vs shorter ones. The association with weight, however, decreased after allowance for height, and no difference was observed for body mass index. Socioeconomic correlates and family history are similar to well-assessed risk factors for female breast cancer. The associations with anthropometric measures and childlessness may find an explanation in chromosomal abnormalities, such as Klinefelter's syndrome, or other hormone-related disorders.


British Journal of Cancer (1995) 71, 1359-1362

© 1995 Stockton Press All rights reserved 0007-0920/95 $12.00

### Risk factors for male breast cancer

B D'Avanzo[1] and C La Vecchia[1,2]

[1]Istituto di Ricerche Farmacologiche 'Mario Negri', Via Eritrea 62, 20157 Milan, Italy; [2]Istituto di Statistica Medica e Biometria, Università di Milano, Via Venezian 1, 20133 Milan, Italy.

**Summary** Risk factors for male breast cancer were investigated in a case-control study of 21 cases and 82 controls admitted to hospital for acute, non-neoplastic, non-hormone-related diseases in the Greater Milan area between 1988 and 1994. More educated men tended to be at higher risk of breast cancer, with a multivariate odds ratio (OR) of 2.6 [95% confidence interval (CI) 0.7-9.4]. The OR was 3.2 (95% CI 1.1-9.6) for those in the higher social class. Men with no offspring were at higher risk than fathers, with an OR of 5.5 (95% CI 1.8-16.7). A history of breast cancer in female relatives was reported by two cases and one control, giving an OR of 8.5 (95% CI 1.1-69.0). Cases were somewhat heavier than controls, and significantly taller, with an OR of 5.7 (95% CI 1.6-19.9) for subjects taller than 170 cm *vs* shorter ones. The association with weight, however, decreased after allowance for height, and no difference was observed for body mass index. Socioeconomic correlates and family history are similar to well-assessed risk factors for female breast cancer. The associations with anthropometric measures and childlessness may find an explanation in chromosomal abnormalities, such as Klinefelter's syndrome, or other hormone-related disorders.

**Keywords:** breast neoplasms; body mass index; infertility; male; social class; case-control study

Male breast cancer is extremely rare, representing less than 1% of all breast cancer and less than 0.1% of all cancer deaths in men (La Vecchia *et al.*, 1992; Sasco *et al.*, 1993). Consequently, epidemiological data are scant. Some associations have been suggested with sociodemographic characteristics, including a direct gradient with social class (i.e. an increased risk in higher social classes) (Sasco *et al.*, 1993), although this remains controversial (Lenfant-Pejovic *et al.*, 1990; Thomas *et al.*, 1992): with marital status, never married men being more frequently affected; and with religion, Jewish men being at highest risk (Mabuchi *et al.*, 1985; Thomas *et al.*, 1992; Sasco *et al.*, 1993).

Anthropometric characteristics have been investigated, and body mass index was associated with male breast cancer risk in a case-control study from Los Angeles County (Casagrande *et al.*, 1988).

Previous breast or testicular disease and gynaecomastia have been related to male breast cancer, and associations are reported with orchiectomy, orchitis, testicular injury, late puberty and infertility (Sasco *et al.*, 1993). Klinefelter's syndrome is substantially more common among male breast cancer patients (Casagrande *et al.*, 1988; Sasco *et al.*, 1993). Possibly as a consequence of Klinefelter's syndrome or hormonal abnormalities, infertility and low fertility have also been associated with male breast cancer (Thomas *et al.*, 1992; Sasco *et al.*, 1993). Family history of breast cancer has repeatedly been associated with breast cancer risk in female and in male first-degree relatives (Casagrande *et al.*, 1988; Rosenblatt *et al.*, 1990; Sasco *et al.*, 1993).

In terms of aetiological mechanisms, high oestrogen levels have been reported as a risk factor for male breast cancer (Sasco *et al.*, 1993), and various studies have found higher serum or urinary oestrogen levels in cases than in controls, but not all results were consistent (Calabresi *et al.*, 1976; Ribeiro *et al.*, 1980; Nirmul *et al.*, 1982; Casagrande *et al.*, 1988; Ballerini *et al.*, 1990; Olsson *et al.*, 1990).

To provide further information on this issue, we report here data from a case-control study on male breast cancer conducted in northern Italy.

Correspondence: B D'Avanzo
Received 12 September 1994; revised 19 December 1994; accepted 6 February 1995

### Materials and methods

Data were derived from a case-control study conducted since 1988, based on a network including the major teaching and general hospitals of the Greater Milan area. Trained interviewers identified and questioned male patients with breast cancer and controls among patients admitted to hospital with similar catchment area for acute, non-neoplastic, non-hormone-related diseases. The present analysis was based on data collected until February 1994.

The structured questionnaire included information about sociodemographic and anthropometric characteristics, general lifestyle habits, such as smoking, alcohol and coffee consumption, a problem-oriented medical history, family history of breast and other hormone-related cancers in first-degree female relatives and frequency of consumption of selected indicator foods.

Cases were 21 patients with histologically confirmed incident breast cancer and were aged 34-74 years (median age 60). They had been diagnosed during the year before interview, and were admitted to the National Cancer Institute and the Ospedale Maggiore of Milan, including the four largest teaching and general hospitals in the Greater Milan area.

Controls were 82 subjects aged 31-74 years (median age 59), in hospital for acute, non-neoplastic, non-hormone-related diseases. Of these, 43% were admitted for traumatic diseases, 18% for non-traumatic orthopaedic diseases, 8% for acute surgical conditions and 31% for various other disorders, such as skin, eye, ear, nose and throat conditions.

The distribution of cases and controls according to age is shown in Table I. Over 80% of cases and 90% of controls resided in the same region, Lombardy. Less than 5% of subjects approached for interview (cases and controls) refused to participate.

**Table I** Distribution of 21 cases of male breast cancer and 82 controls according to age, Milan, Italy, 1988-94

| | *Cases* | | *Controls* | |
|---|---|---|---|---|
| *Age group* | *No.* | *%* | *No.* | *%* |
| <45 | 3 | 14.3 | 14 | 17.1 |
| 45-54 | 5 | 23.8 | 18 | 22.0 |
| 55-64 | 6 | 28.6 | 26 | 31.7 |
| 65-74 | 7 | 33.3 | 24 | 29.3 |

Social class was defined using occupation codes, and socioeconomic groups comparable to those of the British Registrar General (Leete and Fox, 1977) were defined. To obtain categories with meaningful numbers, we grouped the six original social classes into two broad categories: the upper one included classes 1, 2, and 3 non-manual, corresponding to professional, managerial and intermediate occupations; the lower one included classes from 3 manual to 5, corresponding to manual skilled, low skilled and unskilled occupations.

*Data analysis*

Odds ratios (ORs), with the corresponding 95% confidence intervals (CIs) (Breslow and Day, 1980) of breast cancer were computed by means of multiple logistic regression equations, including terms for quinquennia of age plus area of residence, number of offspring, family history of breast cancer, social class, and, whenever indicated, height. For multiple levels of exposure, the significance of the linear trend in risk was assessed by comparing the difference between the deviances of the models without and with the term of interest to the chi-square distribution with one degree of freedom (Breslow and Day, 1980).

### Results

Socioeconomic characteristics, number of offspring and family history of breast cancer are considered in Table II. More educated men tended to be at higher risk of breast cancer. Subjects reporting 12 or more years of schooling had an OR of 2.6 (95% CI 0.7–9.4) compared with less educated ones. Cases also differed from controls with reference to social class, subjects of higher social classes having an OR of 3.2 (95% CI 1.1–9.6). Men with no children were at higher risk than fathers, with a more than 5-fold increased risk (OR 5.5, 95% CI 1.8–16.7). This was not accounted for by differences in marital status between cases and controls, since only 10% of cases and 16% of controls were never married. Family history of breast cancer was reported by two cases and one control, giving an OR of 8.5 (95% CI 1.1–69.0).

Anthropometric characteristics are presented in Table III. Cases were somewhat heavier than controls. Compared with subjects weighing less than 70 kg, the OR for men weighing between 70 and 79 kg was 2.1, and for the heaviest group was 3.4. The trend in risk with weight, however, was not significant. Cases also tended to be taller than controls, and only one case (5%) compared with 23 (28%) controls was

**Table II** Distribution of 21 cases of male breast cancer and 82 controls according to sociodemographic factors and family history, and corresponding odds ratios (with 95% confidence intervals, CI), Milan, Italy, 1988–94

| | *Cases* | | *Controls* | | *Odds Ratios*[a] |
|---|---|---|---|---|---|
| | *No.* | *%* | *No.* | *%* | *(95% CI)* |
| Education (years) | | | | | |
| <7 | 7 | 33.3 | 41 | 50.0 | 1[b] |
| 7–11 | 6 | 28.6 | 22 | 26.8 | 1.8 (0.5–6.5) |
| ⩾12 | 8 | 38.1 | 19 | 23.2 | 2.6 (0.7–9.4) |
| $\chi^2_{1\,trend}$ | | | | | 2.17 |
| Social class | | | | | |
| Lower[c] | 8 | 38.1 | 53 | 64.6 | 1[b] |
| Higher | 13 | 61.9 | 29 | 35.4 | 3.2 (1.1–9.6) |
| Children | | | | | |
| Yes | 6 | 33.3 | 57 | 69.5 | 1[b] |
| No | 15 | 66.7 | 25 | 30.5 | 5.5 (1.8–16.7) |
| Family history of breast cancer | | | | | |
| No | 19 | 90.5 | 81 | 98.8 | 1[b] |
| Yes | 2 | 9.5 | 1 | 1.2 | 8.5 (1.1–69.0) |

[a]Estimates from multiple logistic regression equations including terms for age, height and the above variables. [b]Reference category. [c]Including classes from 3 manual to 5 of the British Registrar's General Classification.

**Table III** Distribution of 21 cases of male breast cancer and 82 controls according to anthropometric variables, and corresponding odds ratios (with 95% confidence intervals, CI), Milan, Italy, 1988–94

| | *Cases* | | *Controls* | | *Odds Ratios*[a] |
|---|---|---|---|---|---|
| | *No.* | *%* | *No.* | *%* | *(95% CI)* |
| Weight (kg) | | | | | |
| <70 | 3 | 14.3 | 34 | 41.5 | 1[b] |
| 70–79 | 8 | 38.1 | 25 | 30.5 | 2.1 (0.4–11.1) |
| ⩾80 | 10 | 47.6 | 23 | 28.0 | 3.4 (0.7–15.7) |
| $\chi^2_{1\,trend}$ | | | | | 2.68 |
| Height (cm) | | | | | |
| <165 | 1 | 4.8 | 23 | 28.0 | 1[b] |
| 165–170 | 4 | 19.0 | 33 | 40.2 | |
| ⩾171 | 16 | 76.2 | 26 | 31.7 | 5.7 (1.6–19.9) |
| $\chi^2_{1\,trend}$ | | | | | 9.24* |
| Body mass index ($kg\,m^{-2}$) | | | | | |
| ⩽24.22 | 7 | 33.3 | 28 | 34.1 | 1[b] |
| 24.23–26.50 | 7 | 33.3 | 26 | 31.7 | 1.1 (0.3–3.5) |
| ⩾26.51 | 7 | 33.3 | 28 | 34.1 | 1.0 (0.3–3.3) |
| $\chi^2_{1\,trend}$ | | | | | 0.07 |

[a]Estimates from multiple logistic regression equations including terms for age, parity, family history and social class. [b]Reference category. *$P<0.01$

shorter than 165 cm; 19% of cases and 40% of controls were between 165 and 170 cm, and 76% of cases but only 32% of controls were taller than 170 cm. The OR for the highest group compared with the two lowest ones was 5.7 (95% CI 1.6–19.9). When the two higher groups were separately compared with the first one, the ORs were 3.2 for the second and 12.9 for the third, and the chi-squared for trend was significant. When the risk estimates for height and weight were mutually adjusted, the OR for height was not materially modified (OR = 5.2, 95% CI 1.3–20.4). Conversely, the OR for weight materially decreased after allowance for height, and it was 1.6 (95% CI 0.3–9.5) for individuals weighing 70–79 kg and 1.5 (95% CI 0.3–8.2) for those weighing more than 79 kg. Consequently, body mass index (BMI, Quetelet index, kg $m^{-2}$), was unrelated to the risk of breast cancer.

### Discussion

This study confirms that male breast cancer has several risk factors similar to those well defined for female breast cancer (Boyle, 1988). In particular, high social class indicators, no offspring and family history of breast cancer were related to male breast cancer.

From a methodological viewpoint, it is unlikely that major information bias influenced the present results. Information and recall bias about family history of breast cancer should be limited, because information was related only to first-degree relatives. Possible problems of information bias can be related to anthropometric measures, since self-reported height tends to be systematically overestimated, and weight underestimated (Stewart *et al.*, 1987). It is unlikely, however, that height and weight were systematically and differently reported by cases and controls, and hence that any such potential bias accounts for the strong associations observed. Other variables investigated, including education, occupation and number of offspring, should not be appreciably affected by systematic bias. Selection bias is also unlikely to have noticeably affected the results, since cases and controls were chosen from similar catchment areas and participation was almost complete.

The association of socioeconomic indicators with breast cancer was not accounted for by other identified risk factors. An American study observed an excess of more educated individuals among cases (Mabuchi *et al.*, 1985), but this was not reported in a French–Swiss study (Lenfant-Pejovic *et al.*, 1990), whereas in another American study including 227 cases the only indicator of higher social class significantly related to male breast cancer risk was Jewish religion (Thomas *et al.*, 1992). With reference to females, an Italian study, conducted on females in the same area and with the same methodology of the present study, found a significantly increased risk of about 50% for more educated and higher social class women (La Vecchia *et al.*, 1987).

In relation to anthropometric measures, the association with height was stronger than that with weight, and the latter was largely accounted for by height. This is consistent with the lack of association with BMI, which is a measure of weight uncorrelated to height (Benn, 1971). This result suggests that some chromosomal disorder, such as Klinefelter's syndrome, may be at the base of the association. Subjects with Klinefelter's syndrome tend in fact to be taller (Wilson and Griffin, 1983). Alternatively, high stature may be an indicator of a more affluent diet in infancy and childhood, in agreement with results regarding women (Swanson *et al.*, 1988). This issue of anthropometric factors and male breast cancer is, however, still debated. In a large population-based American study, no trend of increasing risk with height was found (Thomas *et al.*, 1992), and in another American study (Casagrande *et al.*, 1988) body weight was found to be associated with occurrence of male breast cancer. In that study, anyway, cases were only moderately taller than controls, and no reciprocal allowance was made of weight and height.

Being childless was associated with an increased risk. Although most cases and controls were married, controls had significantly more children than cases, suggesting an underlying problem of infertility among cases. In other studies, fatherhood was protective against male breast cancer, and the risk decreased with numbers of children fathered (Thomas *et al.*, 1992; Sasco *et al.*, 1993).

Other, more accurate indicators of Klinefelter's syndrome were not available, preventing a thorough assessment of this issue. Other studies (Schottenfeld *et al.*, 1963; Nadel and Koss, 1967; Harnden *et al.*, 1971; Scheike *et al.*, 1973; Casagrande *et al.*, 1988) investigated sex chromatin positivity and the proportion of positive cases was around 3%. Thus, also in this population only a fraction of cases of male breast cancer should be related to Klinefelter's syndrome, which, in turn, is unlikely to account totally for the associations with childlessness and height that emerged.

Plasma oestradiol is elevated in subjects with Klinefelter's syndrome, and testosterone is low (Wilson and Griffin, 1983). It has also been suggested that higher oestrogen and lower androgen levels may decrease fertility in the general population (Sharpe and Skakkebaek, 1993). This offers a plausible pathological link for the relationship of male breast cancer also for some of the other risk factors, including anthropometric measures and number of children. Body weight is thought to be associated with increased oestrogen levels, which in men are mainly derived from aromatisation of testosterone in the adipose tissue, and some studies found elevated oestrogen levels in males with breast cancer (Calabresi *et al.*, 1976; Ribeiro *et al.*, 1980; Nirmul *et al.*, 1982; Olsson *et al.*, 1990), although the issue is still unsettled (Casagrande *et al.*, 1988).

Although the limited number of cases and the uncertainties in the interpretations preclude definite conclusions, this study provides some support to the indications that male and female breast cancer share at least some aetiological factors. This is also reflected in the biological characteristics of male breast cancer, whose prognosis and survival appear to be similar to its much more common female counterpart (Adami *et al.*, 1989; Levi *et al.*, 1992; Crocetti and Buiatti, 1994).

**Acknowledgements**

This work was conducted within the framework of the CNR (Italian National Research Council) Applied Project 'Clinical Application of Oncological Research' (Contract No. 94.01321.PF39), and with the contributions of the Italian Association for Cancer Research, the Italian League against Tumours, Milan, and Mrs Angela Marchegiano Borgomainerio. The authors thank Mrs Judy Baggott, Mrs Ivana Garimoldi and the GA Pfeiffer Memorial Library staff for editorial assistance.

### References

ADAMI HO, HAKULINEN T, EWERTZ M, TRETLI S, HOLMBERG L AND KARJALAINEN S. (1989). The survival pattern of male breast cancer. An analysis of 1429 patients from the Nordic countries. *Cancer*, **64**, 1177–1182.

BALLERINI P, RECCHIONE C, CAVALLERI A, MONETA R, SACCOZZI R AND SECRETO G. (1990). Hormones in male breast cancer. *Tumori*, **76**, 26–28.

BENN RT. (1971). Some mathematical properties of weight-for-height indices used as measures of adiposity. *Br. J. Prev. Soc. Med.*, **25**, 42–50.

BOYLE P. (1988). Epidemiology of breast cancer. *Baillière's Clin. Oncol.*, **2**, 1–57.

BRESLOW NE AND DAY NE. (1980). *Statistical Methods in Cancer Research*, Vol. 1. *The Analysis of Case–Control Studies*. IARC Scientific Publications No. 32. IARC: Lyon.

CALABRESI E, DE GIULI G, BECCIOLINI A, GIANNOTTI P, LOMBARDI G AND SERIO M. (1976). Plasma estrogens and androgens in male breast cancer. *J. Steroid Biochem.*, **7**, 605–609.

CASAGRANDE JT, HANISCH R, PIKE MC, ROSS RK, BROWN JB AND HENDERSON BE. (1988). A case–control study of male breast cancer. *Cancer Res.*, **48**, 1326–1330.

CROCETTI NM AND BUIATTI E. (1994). Male breast cancer: incidence, mortality and survival rates from an Italian population-based series. *Eur. J. Cancer*, **30A**, 1732–1733.

HARNDEN DG, MACLEAN N AND LANGLANDS AO. (1971). Carcinoma of the breast and Klinefelter's syndrome. *J. Med. Genet.*, **8**, 460–461.

LA VECCHIA C, DECARLI A, PARAZZINI F, GENTILE A, NEGRI E, CECCHETTI G AND FRANCESCHI S. (1987). General epidemiology of breast cancer in Northern Italy. *Int. J. Epidemiol.*, **16**, 347–355.

LA VECCHIA C, LEVI F AND LUCCHINI F. (1992). Descriptive epidemiology of male breast cancer in Europe. *Int. J. Cancer*, **51**, 62–66.

LEETE R AND FOX AJ. (1977). Registrar General's social classes: origins and uses. *Population Trends*, **8**, 1–7.

LENFANT-PEJOVIC M-H, MLIKA-CABANNE N, BOUCHARDY C AND AUQUIER A. (1990). Risk factors for male breast cancer: a Franco-Swiss case-control study. *Int. J. Cancer*, **45**, 661–665.

LEVI F, RANDIMBISON L AND LA VECCHIA C. (1992). Breast cancer survival in relation to sex and age. *Oncology*, **49**, 413–417.

MABUCHI K, BROSS DS AND KESSLER II. (1985). Risk factors for male breast cancer. *J. Natl Cancer Inst.*, **74**, 371–375.

NADEL M AND KOSS LG. (1967). Klinefelter's syndrome and male breast cancer. *Lancet*, **ii**, 366.

NIRMUL D, PEGORARO RJ, JIALAL I, NAIDOO C AND JOUBERT SM. (1982). The sex-hormone profile of male patients with breast cancer. *Br. J. Cancer*, **48**, 423–427.

OLSSON H, ALM P, ASPEGREN K, GULLBERG B, JÖNSSON PE AND RANSTAM J. (1990). Increased plasma prolactin levels in a group of men with breast cancer. A preliminary study. *Anticancer Res.*, **10**, 59–62.

RIBEIRO GG, PHILLIPS HV AND SKINNER LG. (1980). Serum oestradiol-17$\beta$, testosterone, luteinizing hormone and follicle-stimulating hormone in males with breast cancer. *Br. J. Cancer*, **41**, 474–477.

ROSENBLATT KA, THOMAS DB, JIMENEZ IM, MCTIERNAN A, STALSBERG H, STEMHAGEN A, THOMPSON WD, CURNEN M, SATARIANO A, AUSTIN DF, ISACSON P, GREENBERG RS, KEY CR, KOLONEL L AND WEST D. (1990). Exposure to ionizing radiation and breast cancer in men. *Am. J. Epidemiol.*, **132**, 776.

SASCO J, LOWENFELS AB AND PASKER-DE JONG P. (1993). Review article: Epidemiology of male breast cancer. A meta-analysis of published case–control studies and discussion of selected aetiological factors. *Int. J. Cancer*, **53**, 538–549.

SCHEICKE O, VISFELDT J AND PETERSEN B. (1973). Male breast cancer. 3. Breast carcinoma in association with the Klinefelter syndrome. *Acta Pathol. Microbiol. Scand. A.*, **81**, 352–358.

SCHOTTENFELD D, LILIENFELD AM AND DIAMOND H. (1963). Some observations on the epidemiology of breast cancer among males. *Am. J. Public Health*, **53**, 890–897.

SHARPE RM AND SKAKKEBAEK NE. (1993). Are oestrogens involved in falling sperm counts and disorders of the reproductive tract? *Lancet*, **341**, 1392–1395.

STEWART AW, JACKSON RT, FORD MA AND BEAGLEHOLE R. (1987). Underestimation of relative weight by use of self-reported height and weight. *Am. J. Epidemiol.*, **125**, 122–126.

SWANSON CA, JONES DY, SCHATZKIN A, BRINTON LA AND ZIEGLER RG. (1988). Breast cancer risk assessed by anthropometry in the NHANES I epidemiological follow-up study. *Cancer Res.*, **48**, 5363–5367.

THOMAS DB, JIMENEZ LM, MCTIERNAN A, ROSENBLATT K, STALSBERG H, STEMHAGEN A, THOMPSON WD, MCCREA CURNEN MG, SATARIANO W, AUSTIN DF, GREENBERG RS, KEY C, KOLONEL LN AND WEST DW. (1992). Breast cancer in men: risk factors with hormonal implications. *Am. J. Epidemiol.*, **135**, 734–748.

WILSON JD AND GRIFFIN JE. (1983). Disorders of sexual differentiation. In *Harrison's Principles of Internal Medicine*, 10 ed, Petersdorf RG, Adams RD, Braunwald E, Isselbacher KJ, Martin JB and Wilson JD (eds) pp. 724–739. McGraw-Hill: Auckland.